# Effects of a novel magnetic orthopedic appliance (MOA-III) on the dentofacial complex in mild to moderate skeletal class III children

**DOI:** 10.1186/s13005-015-0092-7

**Published:** 2015-10-14

**Authors:** Ning Zhao, Jing Feng, Zheng Hu, Rongjing Chen, Gang Shen

**Affiliations:** Department of Orthodontics, Shanghai Key Laboratory of Stomatology, Shanghai No. 9 Hospital, ShanghaiJiaotong University School of Medicine, Shanghai, China

**Keywords:** Magnetic, Twin-block, Angle Class III, Adolescent

## Abstract

**Introduction:**

The objective of this study was to evaluate the changes of skeletal and dental structures in mild to moderate skeletal Class III children following the use of a new magnetic orthopedic appliance (MOA-III).

**Methods:**

A total of 36 patients (14 boys and 22 girls, mean age 9 years and 5 months) who presented with a mild to moderate skeletal Class III jaw discrepancy were treated with MOA-III. Another group of 20 untreated patients (9 boys and 11 girls, mean age 9 years and 2 months) with the same level of deformity served as the control group. The average treatment time was 6.6 months. Radiographs were taken at the same time intervals for both groups. A paired *t* test was used to determine the significant differences before and after treatment, and a two-sample *t* test was used to analyze the differences between the treatment and control groups.

**Results:**

The anterior crossbite in all subjects was corrected after MOA-III therapy. The maxillomandibular relationship showed favorable changes (ANB, Wits, overjet increased significantly, *P* < 0.001). The maxilla was anteriorly positioned (SNA, ptm-A, ptm-S increased significantly, *P* < 0.001) with clockwise rotation (PP-FH increased, *P* < 0.001). The mandible showed a slight downward and backward rotation (SNB decreased, *P* < 0.05, MP-SN, Y-axis increased, *P* < 0.05). The length of the mandibular body showed no significant changes (Go-Pg, *P* > 0.05). Significant upper incisor proclination and lower incisor retroclination were observed (UI-NA increased, *P* < 0.001, LI-NB, FMIA decreased, *P* < 0.001). The upper lip moved forward, and the lower lip moved backward (UL-EP increased, *P* < 0.001, LL-EP decreased, *P* < 0.05). In the control group, most of the parameters showed normal growth, except for some unfavorable mandibular skeletal and soft tissue changes (Go-Pg, Go-Co, MP-SN, N′-SN-Pg′ increased, *P* < 0.001). Significant positive changes were induced with the MOA-III appliance compared to the untreated group.

**Conclusions:**

The MOA-III was effective for the early treatment of a mild to moderate Class III malocclusion in children.

## Introduction

Skeletal Class III anomalies are associated with maxillary retrusion, mandibular protrusion, or both. In growing children, treatment may involve the stimulation of maxillary growth and restriction of mandibular growth by orthopedic forces.

Among the armamentarium for the early treatment of class III malocclusion, the chin cup, facemask, and reverse pull headgear are classical orthopedic appliances [1]. However, these appliances need an extraoral apparatus to create heavy orthopedic forces. These appliances are not convenient for patients to wear, and the patients cannot usually guarantee that they will wear them for a sufficient period of time because of their aesthetics. Therefore, the development of new types of intraoral orthopedic appliances to resolve these problems is necessary.

With the introduction of high energy rare earth permanent magnets in the late fifties and early sixties (SmCO_5_, Sm_2_Co_17_) [2], the application of small magnets to create sufficiently high orthopedic forces in the limit-spaced oral cavity became possible. Neodymium iron boron (Nd_2_Fe_14_B) is a new generation high energy rare earth permanent magnet with a high magnetic flux density in relation to its small size. Because of the characteristics of magnetic forces, magnets became another choice to produce the predictive forces used in the field of orthodontics. Blechman and Smiley [3] first moved canines distally using magnetic forces in a cat model in 1978, and since then, magnets have been used in both research and clinical practice. Attractive magnetic forces have been used in closing the diastemas [4], dealing with unerupted or impacted teeth [5–7], intruding posterior teeth [8, 9], moving teeth [10], and manufacturing magnetic edgewise brackets [11]. They were also incorporated into several functional appliances [12–16] to produce orthopedic forces. Repulsive magnetic forces were used for molar distalization [17–19] and palatal expansion [20, 21], and some appliances were used for the treatment of an open bite [22–25] or Class III malocclusion [26].

In this study, we developed a new magnetic orthopedic appliance (MOA-III) using attractive forces at our University. The objective of this study was to examine the craniofacial and dentoalveolar changes in subjects with mild to moderate skeletal class III malocclusion after treatment with this appliance.

## Methods

### Appliance design

**Fig. 1:**
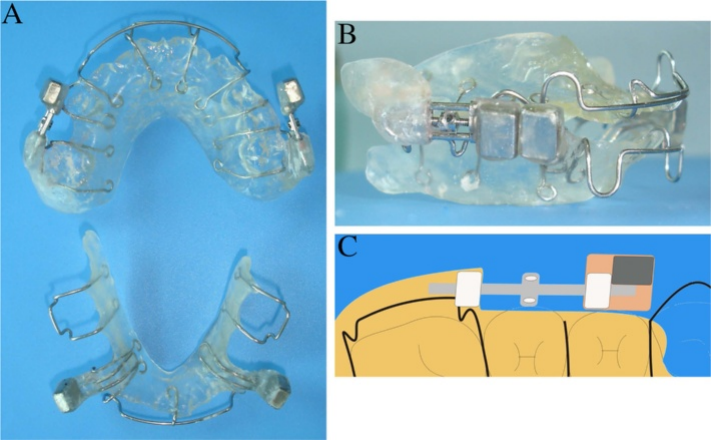
Upper and lower appliance of MOA (**a**), Attractive configuration of magnets extraorally (**b**), Schematic view of appliance of MOA (**c**)

**Fig. 2:**
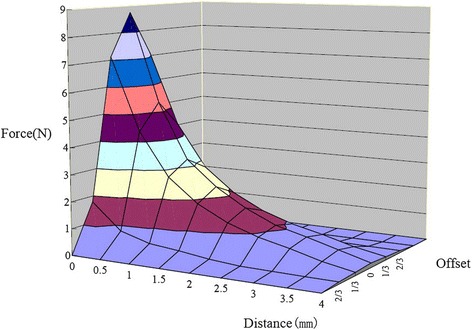
Attractive forces produced between two 7 × 5 × 4 mm^3^ Nd_2_Fe_14_B magnets

The MOA-III appliance was constructed from upper and lower removable appliances with two 7 × 5 × 4 mm^3^ Nd_2_Fe_14_B magnetic units bonded to each appliance (Fig. 1). The two magnetic units were in the attracting configuration. Figure 2 shows the relationship between the forces and distances with 5 × 4 mm^2^ interfaces overlapped, with 1/3 offset and 2/3 offset. The upper magnets were located at the position of the first premolar and bonded to the appliance with two expansion screws, and the lower magnets were positioned labially to the lower canine. The expansion screws were opened to maximum when the appliances were manufactured. After insertion of the MOA-III, the appliances were adjusted by closing the screws to maintain the distances between the paired magnets on both sides. The initial force was 300 g per side when the patients were at the maximal mouth closure position and the two opposing magnets were approximately 1.2 mm apart. The directions of forces were parallel to the occlusal plane. The magnets were conformal coated with Parylene C and encapsulated in dental acrylic, and the opposing poles were covered with a thin layer of acrylic (approximately 0.3-mm thickness). The patients were recalled for an examination two weeks after the first MOA-III delivery. The appointment intervals then were adjusted to four weeks, and screw reactivations were performed by parents one turn each week (0.25 mm/week).

### Case selection

A total of 36 patients (14 boys and 22 girls) complaining of concave profiles or prominent lower jaws by their parents or themselves were included in this study. Their ages ranged from 7.9 to 11.6 years of age, and the average age was 9.5 years (the mean treatment period was 6.6 months). Another 20 patients (9 boys and 11 girls) without treatment served as the control group. Their ages ranged from 7.6 to 11.2 years of age, and the average age was 9.2 years. In most cases, the treatment was postponed for the control group due to the presence of primary molars. The patients in this group were informed that they would receive their treatment after six months. The selection of the cases (treatment and control groups) was based on the following criteria: ① 0°›ANB›-3°; ②Wits distance‹0 mm; ③Angle`s class III molar relationship with anterior cross-bite; ④ with some anterior dental compensation, the upper incisor proclined labially and the lower incisor retroclined lingually, but there were no obviously transverse discrepancies and no need of maxillary expansion; ⑤ the patients could not retrude to edge to edge; and ⑥ without cleft palate or craniofacial syndrome.

Intraoral and extraoral pictures, model casts, and standardized panoramic and cephalometric radiographs were taken at the same time intervals for both groups.

The ethics committee of Ninth People’s Hospital affiliated to Shanghai Jiao Tong University, School of Medicine (Reference No: HE25MAR2012-D03326) approved this study. The treatment procedure in this research met the WMA Declaration of Helsinki - Ethical Principles for Medical Research Involving Human Subjects.

Written informed consent for all participants in this study was obtained from the patients and their parents or guardians. The photo release letters were signed by the parents of the patients presented in this paper.

### Radiograph method

#### Cephalometric measurement

**Fig. 3:**
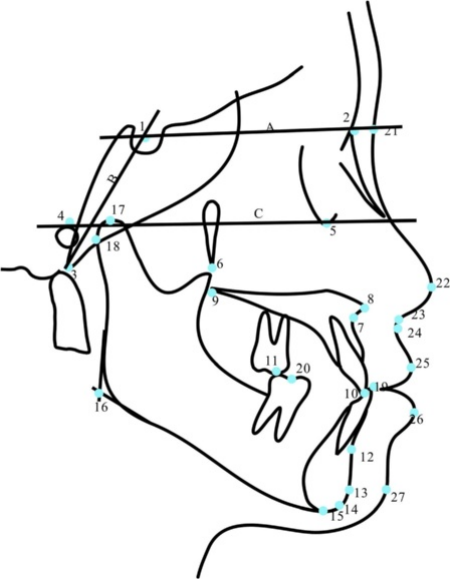
Reference points and reference lines. Reference points: (1) Sella (S), (2) Nasion (N), (3) Basion (Ba), (4) Porion (P), (5) Orbitale (Or), (6) Pterygomaxillary fissure (Ptm), (7) Point A (A), (8) anterior nasal spine (ANS), (9) Posterior nasal spine (PNS), (10) Upper incisor (UI), (11) Upper first molar (U6), (12) Point B, (13) Pogonion (Pog), (14) Gnathion (Gn), (15) Menton (Me), (16) Gonion (Go), (17) Condylion (Co), (18) Articulare (Ar), (19) Lower incisor (LI), (20) Lower first molar (L6), (21) Nasion of soft tissue (N′), (22) Pronasale (Prn), (23) Subnasale (Sn), (24) Point A of soft tissue (A′), (25) Upper labrale (UL), (26) Lower labrale (LL), and (27) Pogonion of soft tissue (Pg′). Reference lines of hard the tissues: (A) Anterior cranial base plane (SN), (B) Cranial base plane (N-Ba), and (C) Frankfort horizontal plane (FH)

We selected 40 sagittal and vertical measurements for the maxillomandibular relationship, maxillary skeletal changes, maxillary dental changes, mandibular dental changes, mandibular skeletal changes and soft tissue changes to determine the dentofacial effects created by the MOA-III treatment. The reference points and lines are shown in Fig. 3, and the cephalometric measurements are shown in Table 1.

**Table 1:** Cephalometric changes between pre- and posttreatment

			Differences	SD	Significance
Maxillomandibular relationship	Saggital	ANB(dg)	2.281	0.789	0.000***
		Wits(mm)	2.394	0.825	0.000***
		overjet(mm)	3.000	0.658	0.000***
	Vertical	FMA(dg)	1.156	1.468	0.007**
		Y-axis(dg)	1.243	1.162	0.001***
		ANS-Me(mm)	−0.518	1.587	0.308 NS
		S-Go(mm)	1.574	1.448	0.001***
		S-Go/N-Me	0.007	0.010	0.014*
		ANS-Me/N-Me	−0.007	0.010	0.014*
		overbite(mm)	0.600	0.572	0.001***
Maxillary skeletal changes	Saggital	SNA(dg)	1.887	0.840	0.000***
		ptm-A(mm)	1.356	0.765	0.000***
		ptm-S(mm)	1.012	0.637	0.000***
	Vertical	N-ANS(mm)	0.950	0.346	0.000***
		PP-FH(dg)	2.381	0.658	0.000***
		OP-SN(dg)	0.962	1.163	0.005***
Changes in maxillary dentition	Saggital	UI-NA(dg)	2.493	1.647	0.000***
		UI-NA(mm)	1.637	1.067	0.000***
		UI-AP(mm)	1.606	0.715	0.000***
		U6-PTM(mm)	1.100	1.130	0.001***
		UI-SN(dg)	3.123	1.569	0.000***
	Vertical	UI-PP(mm)	0.416	0.413	0.002**
		U6-PP(mm)	−0.412	0.923	0.094 NS
Changes in mandibular dentition	Saggital	LI-NB(dg)	−0.743	0.511	0.000***
		LI-NB(mm)	−0.975	0.425	0.000***
		LI-AP(mm)	−1.731	0.967	0.000***
		FMIA(dg)	−1.987	0.770	0.000***
	Vertical	LI-MP(mm)	0.506	1.021	0.066 NS
		L6-MP(mm)	0.075	1.096	0.788 NS
Mandibular skeletal changes	Saggital	SNB(dg)	−0.494	0.843	0.033*
		Go-Pg(mm)	0.781	1.957	0.131 NS
		Pcd-S(mm)	0.350	0.603	0.035*
	Vertical	Go-Co(mm)	0.762	0.396	0.000***
		MP-SN(dg)	1.175	1.064	0.001***
		Y-axis(dg)	1.256	1.400	0.003**
Soft tissue changes		UL-EP(mm)	1.312	0.602	0.000***
		LL-EP(mm)	−1.575	1.498	0.001***
		Z-angel(dg)	1.238	3.086	0.130 NS
		UL-A′-FH(dg)	1.875	2.513	0.009**
		N′-Pg′-FH(dg)	−2.056	2.134	0.002**
		N′-SN-Pg′(dg)	−4.586	1.333	0.000***

NS indicates nonsignificance

*P < .05. **P < .01. ***P < .001

### Statistical methods and method error (ME) analysis

The error of the stated and calculated method values were determined by retracing the radiographs with Dahlberg’s formula, $\mathrm{ME}\kern0.5em =\kern0.5em \sqrt{\frac{{\displaystyle \sum {d}^2}}{2n}}$. The cephalometric radiographs were traced and evaluated twice by two independent orthodontists (N Z and Z H) on two separate occasions approximately two months apart. There were no significant differences between the two repeated measurements at the two assessment times (*P* > 0.05).

Paired t tests were performed with SPSS (Statistical Package for Social Sciences, Chicago, Illinois, USA) 15.0 for Windows to evaluate the significant differences between the pre- and the post-treatment groups and changes in the control group. Two-sample t tests were used to detect significant differences between the two groups by comparing the treatment-induced changes versus the growth-only-induced changes. The level of significance was set at *P* < 0.05.

## Results

### Occlusal changes

**Fig 4:**
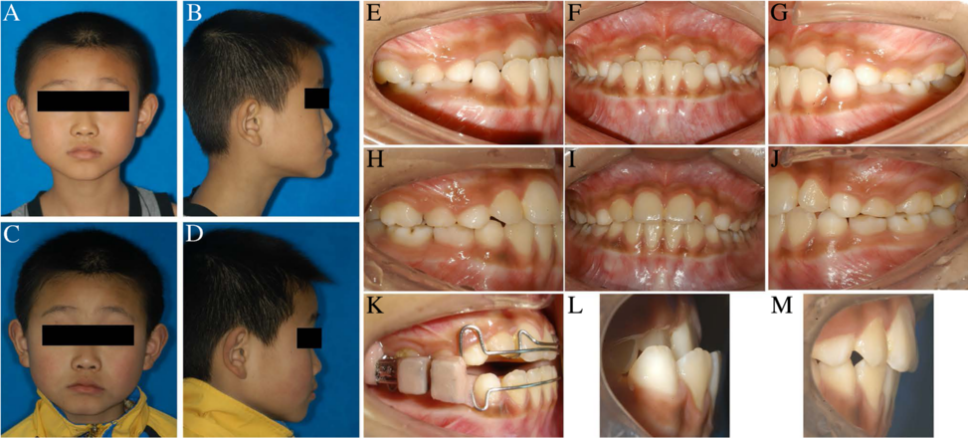
A case treated with MOA-III (comparison of pre- and post-treatment). Pretreatment: Extraoral photos (**a**, **b**), intraoral photos (**e**, **f**, **g**), overbite/overjet (**l**). Posttreatment: Extraoral photos (**c**, **d**), intraoral photos (**h**, **i**, **j**), overbite/overjet (**m**). Intreatment: intraoral photos (**k**)

In the MOA-III treatment group, the anterior crossbite in all subjects was corrected (the mean treatment period was 6.6 months). Class III molar relationships were changed to class I in 32 of 36 patients and were improved in others. We reported one case to show the effects between pre- and post-treatment (Fig. 4). In the control group, all of the patients continued to demonstrate a Class III molar relationship.

### Cephalometric changes (Table 1)

In the maxilla, sagittally, many measurements were significantly increased as follows: ANB (*P* < 0.001), Wits (*P* < 0.001), overjet (*P* < 0.001). SNA (*P* < 0.001), Ptm-A (*P* < 0.001), Ptm-S (*P* < 0.001) and N-ANS (*P* < 0.001). Vertically, the FMA (*P* < 0.001) and Y-axis angle (*P* < 0.001) increased significantly. The overbite deepened significantly (*P* < 0.01), but the ANS-Me (*P* > 0.05), ANS-Me/N-Me (*P* > 0.05) and S-Go/N-Me (*P* > 0.05) showed no significant changes. The PP-FH (*P* < 0.001) and OP-SN (*P* < 0.001) were rotated clockwise significantly. The upper incisors and molars also showed some significant changes. The UI-NA (*P* < 0.001) and UI-AP (*P* < 0.001) increased significantly. The upper first molar moved forward significantly, with U6-PTM (*P* < 0.01). Vertically, the UI-PP (*P* < 0.01) increased significantly, but the U6-PP showed no significant difference (*P* > 0.05). For the lower, the SNB angle (*P* < 0.001) and Pcd-S (*P* < 0.001) significantly decreased. The length of the mandibular body showed no significant changes (Go-Pg, *P* > 0.05). However, the length of the mandibular ramous showed a significant increase (Go-Co, *P* < 0.001). The LI-NB (*P* < 0.001), LI-AP (*P* < 0.001), and FMIA (*P* < 0.001) decreased significantly, although there were no significant changes in the LI-MP (*P* > 0.05) and L6-MP (*P* > 0.05). Much of the soft tissue also showed significant changes. The UL-EP (*P* < 0.001), LL-EP (*P* < 0.01), and UL-A′-FH (*P* < 0.01) increased significantly. The LL-EP (*P* < 0.01) and N′-Sn-Pg′ (*P* < 0.001) decreased significantly. The Z-angle (*P* > 0.05) showed no significant difference.

In the control group, all of the patients continued to demonstrate a class III molar relationship, and most of the cephalometric measurements showed no significant difference. However, the mandibular skeletal measurements and soft tissue measurement showed significant changes. The SNB、Go-Pg、and Pcd-S showed a significant increase (*P* < 0.05). For the soft tissue measurement, the LL-EP, UL-A′-FH, N′-Pg′-FH, and N′-SN-Pg′ increased (*P* < 0.01) and the UL-EP decreased (*P* < 0.01) (Table 2). Comparison of the treated and untreated control group showed that ANB, Wits, overjet, Ptm-A, N-ANS, PP-FH, UI-NA, LI-NB, LI-AP, FMIA, UL-EP, and N′-SN-Pg′changed significantly (*P* < .001) (Table 3).

**Table 2:** Cephalometric changes in untreatment group

			Differences	SD	Significance
Maxillomandibular relationship	Saggital	ANB(dg)	−0.113	0.146	0.065 NS
		Wits(mm)	0.112	0.216	0.185 NS
		overjet(mm)	−0.107	0.189	0.152 NS
	Vertical	FMA(dg)	0.062	0.417	0.684 NS
		Y-axis(dg)	0.100	0.141	0.085 NS
		ANS-Me(mm)	0.200	0.634	0.402 NS
		S-Go(mm)	0.200	0.160	0.010*
		S-Go/N-Me	−0.001	0.002	0.906 NS
		ANS-Me/N-Me	0.000	0.002	0.906 NS
		overbite(mm)	−0.037	0.199	0.611 NS
Maxillary skeletal changes	Saggital	SNA(dg)	0.400	0.523	0.067 NS
		ptm-A(mm)	0.312	0.083	0.036*
		ptm-S(mm)	0.225	0.128	0.002**
	Vertical	N-ANS(mm)	0.337	0.856	0.302 NS
		PP-FH(dg)	0.087	0.339	0.490 NS
		OP-SN(dg)	0.100	0.277	0.342 NS
Changes in maxillary dentition	Saggital	UI-NA(dg)	0.137	0.388	0.350 NS
		UI-NA(mm)	0.037	0.272	0.708 NS
		UI-AP(mm)	−0.142	0.440	0.700 NS
		U6-PTM(mm)	0.312	0.318	0.027*
		UI-SN(dg)	0.200	0.325	0.125 NS
	Vertical	UI-PP(mm)	0.025	0.401	0.143 NS
		U6-PP(mm)	0.137	0.184	0.073 NS
Changes in mandibular dentition	Saggital	LI-NB(dg)	−0.087	0.294	0.429 NS
		LI-NB(mm)	−0.213	0.314	0.096 NS
		LI-AP(mm)	−0.163	0.272	0.135 NS
		FMIA(dg)	−0.25	0.239	0.021*
	Vertical	LI-MP(mm)	0.187	0.325	0.064 NS
		L6-MP(mm)	0.175	0.211	0.310 NS
Mandibular skeletal changes	Saggital	SNB(dg)	0.363	0.082	0.003**
		Go-Pg(mm)	0.462	0.106	0.001**
		Pcd-S(mm)	0.212	0.124	0.002**
	Vertical	Go-Co(mm)	0.400	0.220	0.001***
		MP-SN(dg)	0.237	0.106	0.000***
		Y-axis(dg)	0.150	0.277	0.170 NS
Soft tissue changes		UL-EP(mm)	−0.225	0.116	0.001***
		LL-EP(mm)	0.250	0.141	0.002**
		Z-angel(dg)	−0.225	0.128	0.002**
		UL-A′-FH(dg)	0.025	0.362	0.850 NS
		N′-Pg′-FH(dg)	0.375	0.183	0.001***
		N′-SN-Pg′(dg)	0.375	0.070	0.000***

NS indicates nonsignificance

*P < .05. **P < .01. ***P < .001

**Table 3:** Changes between treatment and control group

			Differences	SD	Significance
Maxillomandibular relationship	Saggital	ANB(dg)	2.394	0.843	0.000***
		Wits(mm)	2.282	0.925	0.000***
		overjet(mm)	3.107	0.798	0.000***
	Vertical	FMA(dg)	1.094	1.557	0.013*
		Y-axis(dg)	1.143	1.349	0.007**
		ANS-Me(mm)	−0.718	1.574	0.057 NS
		S-Go(mm)	1.374	1.727	0.003**
		S-Go/N-Me	0.008	0.017	0.352 NS
		ANS-Me/N-Me	−0.007	0.016	0.304 NS
		overbite(mm)	0.637	0.651	0.005**
Maxillary skeletal changes	Saggital	SNA(dg)	1.487	0.935	0.000***
		ptm-A(mm)	1.044	0.740	0.000***
		ptm-S(mm)	0.787	0.835	0.005**
	Vertical	N-ANS(mm)	0.613	0.366	0.000***
		PP-FH(dg)	2.294	0.662	0.000***
		OP-SN(dg)	0.862	1.219	0.012 NS
Changes in maxillary dentition	Saggital	UI-NA(dg)	2.356	1.631	0.000***
		UI-NA(mm)	1.6	1.039	0.000***
		UI-AP(mm)	1.748	0.697	0.000***
		U6-PTM(mm)	0.788	1.179	0.015**
		UI-SN(dg)	2.923	1.524	0.000***
	Vertical	UI-PP(mm)	0.391	0.359	0.002**
		U6-PP(mm)	−0.549	0.892	0.108 NS
Changes in mandibular dentition	Saggital	LI-NB(dg)	−0.656	0.522	0.000***
		LI-NB(mm)	−0.762	0.421	0.000***
		LI-AP(mm)	−1.568	0.964	0.000***
		FMIA(dg)	−1.737	0.841	0.000***
	Vertical	LI-MP(mm)	0.319	1.076	0.243 NS
		L6-MP(mm)	−0.1	1.226	0.854 NS
Mandibular skeletal changes	Saggital	SNB(dg)	−0.857	0.813	0.072 NS
		Go-Pg(mm)	0.319	2.265	0.188 NS
		Pcd-S(mm)	0.138	0.577	0.050 NS
	Vertical	Go-Co(mm)	0.362	0.843	0.057*
		MP-SN(dg)	0.938	1.095	0.005**
		Y-axis(dg)	1.106	1.349	0.007**
Soft tissue changes		UL-EP(mm)	1.537	0.657	0.000***
		LL-EP(mm)	−1.825	1.522	0.004**
		Z-angel(dg)	1.463	3.281	0.270 NS
		UL-A′-FH(dg)	1.85	2.640	0.016 NS
		N′-Pg′-FH(dg)	−2.431	2.000	0.005**
		N′-SN-Pg′(dg)	−4.961	1.303	0.000***

NS indicates nonsignificance

*P < .05. **P < .01. ***P < .001

## Discussion

Rare-earth magnets, which generate static magnetic fields, have been advantageously used as a 'force source' in orthodontic treatments, such as molar distalization, palatal expansion, and impacted tooth movement [4–10]. There is little evidence regarding the biological safety of static magnetic field application. Some studies suggested that static magnetic fields may increase the rate of bone repair [35] and new bone deposition [33] and may also prevent decreases in bone mineral density caused by surgical invasion or implantation [36]. Bondemark demonstrated that there was no difference between test and control tissues in human buccal mucosa, except for some contact irritation. An overview of rare earth magnets used in orthodontics by Noar [37] suggested that neodymium-iron-boron magnets must be coated with a substance when they are used in the oral environment. In this study, the magnets were conformal coated with Parylene C and encapsulated in dental acrylic.

**Fig 5:**
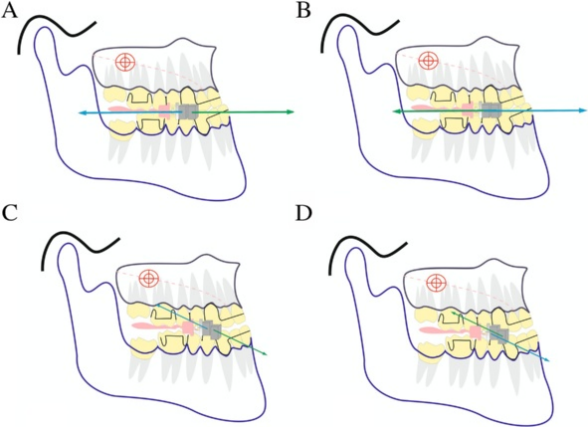
Diagram of the force direction created by MOA-III using repelling/attractive magnetic forces when the patients were at maximal mouth closure and the opening position. **a**: Repelling force at maximal mouth closure. **b**: Attractive force at maximal mouth closure. **c**: Repelling force at the mouth opening position (1/3 offset for opposing magnets). **d**: Attractive force at the mouth opening position (1/3 offset for opposing magnets)

In our previous study, Xu [26] developed a type of magnetic twin-block appliance (TMA) using repelling magnetic forces for the treatment of early skeletal class III malocclusion. This pilot study presented favorable results in growing subjects. However, there were also several unfavorable effects, such as a counter clockwise rotation of the palatal plane and clockwise rotation of the mandibular plane. We also investigated the effects of repelling magnetic orthopedic forces in rhesus monkeys [27] and showed the same advantages and disadvantages as TMA treatment. This phenomenon may be because the force vector in the maxillary magnets is divided into forward and upward components, whereas the forces in the lower magnets are divided into backward and downward components when the patient opens their mouth during masticating activities or at rest (Fig. 5a, c).

Therefore, in this study, we modified the MOA-III appliance to overcome these disadvantages by using attractive forces. When the patients open their mouth while they are speaking, masticating, or performing other oral activities, the attracting magnets create downward and forward force vectors in the upper area and backward and upward vectors in the lower area. The force applied on the maxilla passes near the maxillary center of resistance and may reduce some of the anticlockwise rotations caused by other orthopedic appliances because the center of resistance for a maxilla is slightly inferior to the orbital for the maxilla. By contrast, the force on the lower jaw passed near the center of the condyle, which led to the restraint of mandibular growth (Fig. 5b, d). This intermaxillary force system could be resolved into horizontal, vertical, and transverse components. The horizontal vector pushes the maxilla forward and constrains the lower jaw in an advanced sagittal posture. The vertical forces pull the appliances together and encourage the patients to actively occlude. The transverse components could restrain some lateral mandibular movements. A distinctive aspect of this appliance is the placement of reverse screw expansioners that secure constant magnetic forces by maintaining an adequate distance between the attractive magnets.

During the early treatment of Class III malocclusion, several types of magnetic appliances were developed in clinic and animal studies. Vardimon and co-workers [14, 15] developed Functional Orthopedic Magnetic appliances (FOMA III) and found that the cumulative protraction of the maxillary complex was initiated at the pterygomaxillary fissure, with an additional contribution provided by other circumaxillary sutures, and that the inhibition of mandibular length was minimal in monkeys. Darendeliler [13] used a Magnetic Expansion Device (MED) in conjunction with the MAD III appliance for the early treatment of a Class III malocclusion. After removal of the appliances, the patient showed a Class I dental relationship, with an adequate overjet and overbite and no crossbite. Xu [26] developed a type of magnetic twin-block appliance (TMA) that corrected the Class III molar relationship to Class I in growing subjects with skeletal Class III malocclusion. Tuncer [28] used a magnetic appliance in the treatment of functional Class III patients. The results indicate that the primary effect of this magnetic appliance was an increase in the posterior rotation of the mandible. In our study, the changes in the maxilla were the most important factors contributing to the treatment effect. Maxillary skeletal and dental changes in the anteroposterior direction were evidenced by the forward movement of the A point together with increases of SNA, ptm-A, ptm-S, UI-NA, UI-AP, and U6-Ptm. This was similar to other studies using a protraction facemask with or without maxillary expansion [29]. In the vertical dimension, increasing the N-ANS and clockwise rotations of the palatal plane and occlusion plane may be caused by the downward and forward force components in the upper appliance. In the mandible, the restraint of the lengths of the mandibular body and mandibular ramous were not significant. The increased MP-SN and Y-axis indicated slight downward and backward mandibular rotations. This was similar to the treatment effects of using a chincap [30–32]. Significant changes were found in the lower incisors. The decrease of LI-NB, LI-AP, and FMIA indicated that the lower incisors tipped lingually under the backward forces in the lower appliance. Measurement of the soft tissue showed that the concave profile was improved and that the upper lip moved forward and the lower lip retruded backward. In a randomized controlled trial study using a removable mandibular retractor [34], the main significant findings were similar to our study. For example, there was an anterior morphogenetic rotation of the mandible, a significant increase in maxillary length, a significant increase in maxillary dentoalveolar protrusion, a significant decrease in mandibular dentoalveolar protrusion, a significant protrusion of the upper lip, a significant retrusion of the lower lip, and a significant reduction in the nasolabial angle.

In the untreated control group, the cephalometric measurements indicated that uncontrolled mandibular growth may exaggerate the Class III malocclusion and make the concave profiles worse.

By comparing the results of the MOA-III treatment with normal growth in the untreated Cl III subjects, we could conclude that MOA-III was effective for the treatment of mild skeletal Class III children.

## Conclusion

- MOA-III was effective for the treatment of mild to moderate class III malocclusions in children.
- In the maxilla, both the skeleton and dentition moved forward in the anteroposterior direction. Simultaneously, the maxilla rotated forward and downward. In the mandible, the most significant changes were lingual compensation of the lower incisors. At the same time, the mandible rotated downward and backward, but the length of the mandible body showed no significant changes.
- For the soft tissue measurement, the upper lip moved forward and the lower lip retruded backward. The concave profiles were also improved.
